# Using geographic information systems to identify prospective marketing areas for a special library

**DOI:** 10.1186/1742-5581-3-4

**Published:** 2006-05-04

**Authors:** Rozalynd P McConnaughy, Steven P Wilson

**Affiliations:** 1 School of Medicine Library, University of South Carolina, 6311 Garners Ferry Road, Columbia, SC 29208, USA

## Abstract

**Background:**

The Center for Disability Resources (CDR) Library is the largest collection of its kind in the Southeastern United States, consisting of over 5,200 books, videos/DVDs, brochures, and audiotapes covering a variety of disability-related topics, from autism to transition resources. The purpose of the library is to support the information needs of families, faculty, students, staff, and other professionals in South Carolina working with individuals with disabilities. The CDR Library is funded on a yearly basis; therefore, maintaining high usage is crucial. A variety of promotional efforts have been used to attract new patrons to the library. Anyone in South Carolina can check out materials from the library, and most of the patrons use the library remotely by requesting materials, which are then mailed to them. The goal of this project was to identify areas of low geographic usage as a means of identifying locations for future library marketing efforts.

**Methods:**

Nearly four years worth of library statistics were compiled in a spreadsheet that provided information per county on the number of checkouts, the number of renewals, and the population. Five maps were created using ArcView GIS software to create visual representations of patron checkout and renewal behavior per county.

**Results:**

Out of the 46 counties in South Carolina, eight counties never checked out materials from the library. As expected urban areas and counties near the library's physical location have high usage totals.

**Conclusion:**

The visual representation of the data made identification of low usage regions easier than using a standalone database with no visual-spatial component. The low usage counties will be the focus of future Center for Disability Resources Library marketing efforts. Due to the impressive visual-spatial representations created with Geographic Information Systems, which more efficiently communicate information than stand-alone database information can, librarians may benefit from the software's use as a supplemental tool for tracking library usage and planning promotional efforts.

## Background

The Center for Disability Resources Library (CDR Library) is a special library that serves anyone living in South Carolina, especially professionals who work with individuals with disabilities and family members of children with special needs. The CDR Library is a collaborative effort between BabyNet/South Carolina Department of Health and Environmental Control, the Center for Disability Resources, the South Carolina Department of Disabilities and Special Needs, and the University of South Carolina School of Medicine Library. The CDR Library consists of over 5,200 books, videos, brochures, and audiotapes covering a variety of disability-related topics. Since 2001, the CDR Library has been part of the University of South Carolina School of Medicine Library, which is located in Columbia, South Carolina. The library is funded by a yearly contract; thus, the library's utilization is vital to its existence.

Approximately 651,000 people in South Carolina have a disability [1]. An estimated 108,000 people in South Carolina have difficulty performing self-care activities, such as dressing, bathing, or other activities of daily living. Over 233,000 South Carolinians have a cognitive disability. Most of the information requests received by the CDR Library are related to mental disabilities and children. During the 2001–2002 school year, 110,037 children ages 3 to 21 in South Carolina were served under the Individuals with Disabilities Education Act (IDEA), Part B [2].

The librarians working with the CDR collection have attempted to promote the library and its services in a variety of ways. A monthly newsletter is distributed to patrons via mail and/or email to increase awareness of new resources and to promote the CDR librarians' monthly outreach activities. Librarians also give presentations and tours of the CDR Library to local support groups, University of South Carolina classes, and organizations supporting individuals with disabilities. Other marketing efforts include distributing CDR Library pamphlets by mail, advertising the library in various organizations' newsletters, and participating in live radio spots promoting the collection. The major focus of these efforts in the past has involved promoting the collection to particular groups by exhibiting at various professional conferences instead of publicizing library services to specific areas of the state. Myrtle Beach (Horry County), Charleston (Charleston County), Columbia (Richland County), and Greenville (Greenville County) are common locations for these conferences.

Geographic Information Systems (GIS) can be a very useful problem solving, planning, and service development tool for libraries. GIS has been used by libraries to study community demographics for collection development purposes. One study used demographic data that was visually represented to determine whether to add a consumer health collection in a public library [3]. Librarians have also used GIS to determine the location of new branches by plotting current library locations. In addition to having a visual representation of current library locations, Weber County Library System in northern Utah used GIS to plan the location of a new branch by analyzing patron addresses and demographic data [4]. GIS has also been used to study library book usage with regard to bookshelf heights and spatial distributions of the books [5].

The objective of this study was to identify prime locations for future library promotion efforts by determining which counties in South Carolina were utilizing the Center for Disability Resources Library the least.

## Methods

Nearly four years worth of circulation statistics from June 1, 2001, to February 17, 2005, were compiled by running a report in Innovative Interfaces, the library's integrated library system. Circulation statistics obtained included the number of items checked out and renewed per patron. This information was added to an Excel spreadsheet (Table 1).

**Table 1:** CENTER FOR DISABILITY RESOURCES LIBRARY USAGE STATISTICS:JUNE 1, 2001 – FEBRUARY 17, 2005

**COUNTY**	TOTAL PATRONS*	**TOTAL CHECKOUT**	**TOTAL RENEWAL**	**CENSUS 2000**
ABBEVILLE	1	4	1	26,167
AIKEN	13	240	145	142,552
ALLENDALE	0	0	0	11,211
ANDERSON	17	81	52	165,740
BAMBERG	1	3	2	16,658
BARNWELL	0	0	0	23,478
BEAUFORT	3	10	1	120,937
BERKELEY	2	11	1	142,651
CALHOUN	2	5	2	15,185
CHARLESTON	19	77	24	309,969
CHEROKEE	1	6	1	52,537
CHESTER	3	9	1	34,068
CHESTERFIELD	1	2	2	42,768
CLARENDON	0	0	0	32,502
COLLETON	1	4	4	38,264
DARLINGTON	2	14	4	67,394
DILLON	0	0	0	30,722
DORCHESTER	3	8	7	96,413
EDGEFIELD	0	0	0	24,595
FAIRFIELD	2	14	1	23,454
FLORENCE	4	13	10	125,761
GEORGETOWN	3	17	7	55,797
GREENVILLE	13	65	29	379,616
GREENWOOD	5	68	32	66,271
HAMPTON	1	1	1	21,386
HORRY	7	66	49	196,629
JASPER	0	0	0	20,678
KERSHAW	4	14	4	52,647
LANCASTER	3	9	5	61,351
LAURENS	1	20	0	69,567
LEE	1	2	6	20,119
LEXINGTON	21	92	46	216,014
MARION	1	1	0	35,466
MARLBORO	0	0	0	28,818
MCCORMICK	0	0	0	9,958
NEWBERRY	2	5	9	36,108
OCONEE	5	14	5	66,215
ORANGEBURG	2	4	0	91,582
PICKENS	4	18	11	110,757
RICHLAND	116	959	883	320,677
SALUDA	4	19	18	19,181
SPARTANBURG	8	18	11	253,791
SUMTER	2	81	47	104,646
UNION	1	7	1	29,881
WILLIAMSBURG	2	10	11	37,217
YORK	11	82	18	164,614

* Total Patrons does not include patrons that have only used the library's web resources.

Next, each patron's address was matched to a South Carolina county by using the United States Postal Service Zip Code Lookup tool, which is freely available online [6]. Although the zip code for each patron is included in the address field of his/her patron record, the county is not included. Therefore the zip code lookup tool was used to find out which zip code corresponded to each patron's county. After entering an address and selecting mailing industry information, the USPS web site lists information, including the county of the address. Patron names and addresses were removed from the spreadsheet for confidentiality and replaced by a county name. Population data per county from Census 2000 data was also added to the spreadsheet [7]. The population totals were used to calculate the number of items checked out and renewed per person.

These usage trends were spatially referenced and displayed visually using ArcView GIS (Geographic Information Systems) software. The spreadsheet data was merged with an existing shape file database containing South Carolina county location data.

**Figure 1:**
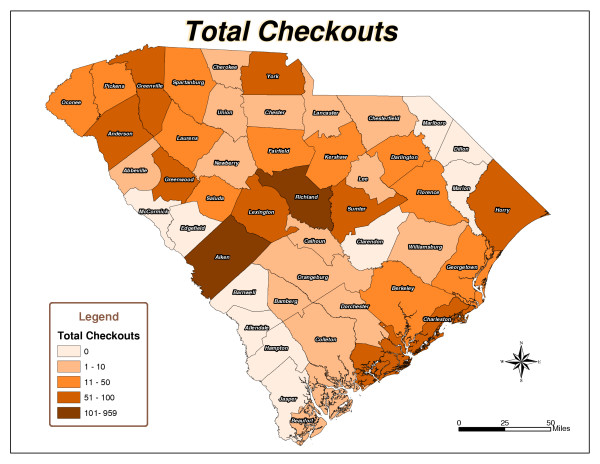
Total Checkouts.

**Figure 2:**
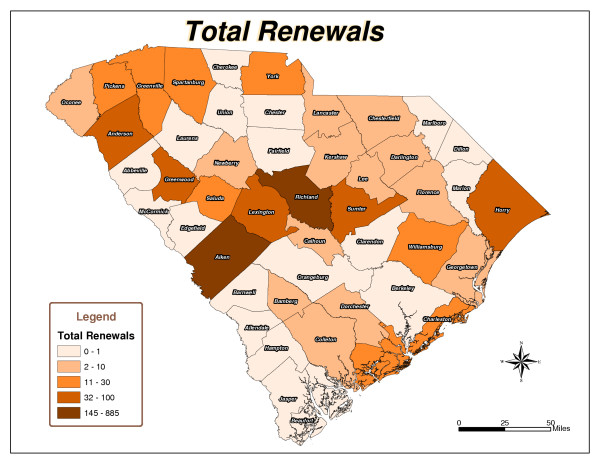
Total Renewals.

**Figure 3:**
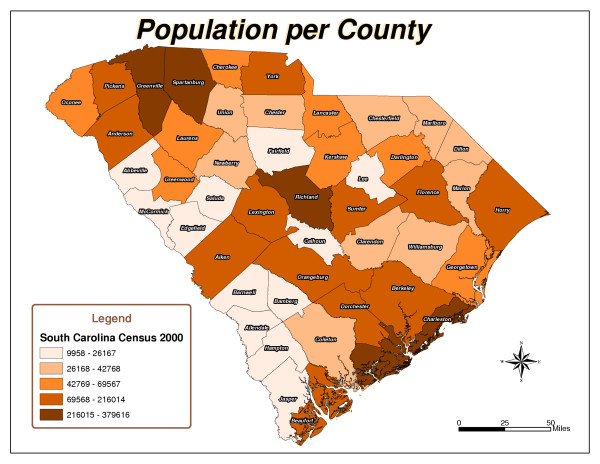
Population Per County.

**Figure 4:**
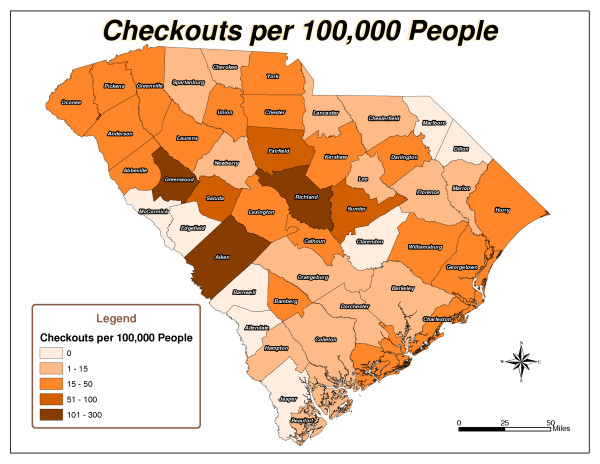
Checkouts Per 100,000 People.

**Figure 5:**
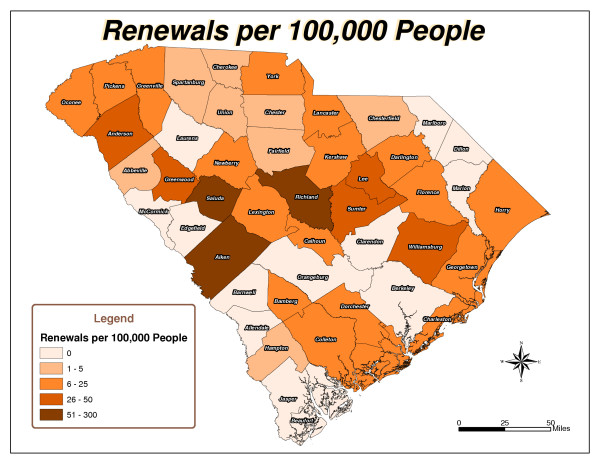
Renewals Per 100,000 People.

Five South Carolina county maps were created: Total Checkouts per County (Figure 1), Total Renewals per County (Figure 2), Population per County (Figure 3), Checkouts per 100,000 people (Figure 4), and Renewals per 100,000 people (Figure 5).

## Results

As expected, urban counties, or those nearest to major metropolitan areas like Aiken, Charleston, Greenville and Richland, and counties closest to the library's physical location had high usage totals. There are 46 counties in South Carolina. The following ten counties had the highest number of items checked out from the library: Aiken, Anderson, Charleston, Greenville, Greenwood, Horry, Lexington, Richland, Sumter, and York (Figure 1). Six counties, including Aiken, Fairfield, Greenwood, Richland, Saluda, and Sumter, had the highest number of checkouts per 100,000 people (Figure 4). Aiken, Anderson, Greenwood, Horry, Lexington, Richland, and Sumter had the highest number of renewals (Figure 2). Eight counties, including Aiken, Anderson, Greenwood, Lee, Richland, Saluda, Sumter, and Williamsburg, had the highest number of renewals per 100,000 people (Figure 5).

The northeast and southwest regions of South Carolina were regions of lower usage. Eight counties, Allendale, Barnwell, Clarendon, Dillon, Edgefield, Jasper, Marlboro, and McCormick, had never borrowed materials from the library (Figure 1). In addition to the counties that had never borrowed materials, Laurens, Marion, and Orangeburg had not renewed library materials (Figure 2). The following fourteen counties also had low per capita checkout rates: Beaufort, Berkeley, Cherokee, Chesterfield, Colleton, Dorchester, Florence, Hampton, Lancaster, Lee, Marion, Newberry, Orangeburg, and Spartanburg (Figure 4). Concerning the counties that had renewed library items, the following ten counties had the lowest number of renewals per 100,000 people: Abbeville, Beaufort, Berkeley, Cherokee, Chester, Chesterfield, Fairfield, Hampton, Spartanburg, and Union (Figure 5).

The maps accurately reflect the use of print and audiovisual materials, but the library usage data does not account for information photocopied or web article links emailed to patrons. Though these services are readily available at the library, the fact that items such as photocopies and web article links cannot be traced back to individual patrons required their exclusion from the data-gathering process, and may potentially threaten the validity of the data obtained.

Other potential confounding variables not discussed in this paper include the number of patrons by county, those patrons who moved between counties or out of the state altogether; the driving distance required of patrons to use the library in person; the relative proportion of special needs individuals by county; and, the relative availability of disability-related materials by public library and each public library's respective location within its county. While it would have been most useful to have a shared legend for comparison purposes, the extreme data distributions required that each map's quintiles be adjusted accordingly.

## Conclusion

Using GIS to identify low usage areas of library materials is an effective means for identifying future marketing areas. The visual representation of the data made identification of low usage regions easier than using a standalone database with no visual-spatial component. Not only could low usage counties be identified, regions of low checkouts and renewals were also evident in the maps. These maps may now be used to communicate visually to the Center for Disability Resources director the need for increased funding for outreach efforts aimed specifically at low usage counties. Moreover, in addition to suggesting counties where new patrons may be solicited, the maps illustrate, literally, those areas with existing users who should continually be encouraged, via marketing and outreach efforts, to take advantage of the library's valuable resources.

There are a number of ways to market the library to new patrons based on location. Since each county has a Disability and Special Needs (DSN) Board that serves individuals with disabilities and their families, CDR Librarians could offer to give presentations to DSN Boards in low usage counties. CDR Librarians may also identify support groups and occupations designed to help individuals with disabilities and their families in these low use areas. Local libraries and relevant businesses could house CDR Library pamphlets or a temporary display about the collection. Future studies using ArcView GIS may determine how successful such marketing efforts are at attracting new patrons to various library collections and services.

## Abbreviations

CDR = Center for Disability Resources

DSN = Disability and Special Needs

GIS = Geographic Information Systems

## Competing interests

The author(s) declare that they have no competing interests.

## Authors' contributions

RPM created the database, the maps, and drafted the manuscript. SPW managed patron usage statistics and identified patron counties. SPW edited and revised the manuscript. All authors read and approved the final manuscript.
